# Protocol for the development of a Core Outcome Set (COS) for hemorrhoidal disease: an international Delphi study

**DOI:** 10.1007/s00384-017-2833-5

**Published:** 2017-05-13

**Authors:** R. R. van Tol, J. Melenhorst, C. D. Dirksen, L. P. S. Stassen, S. O. Breukink

**Affiliations:** 1 Department of Surgery, Maastricht University Medical Centre, P. Debelaan 25, 6229 HX Maastricht, the Netherlands; 2 Department of Clinical Epidemiology and Medical Technology Assessment, Maastricht University Medical Centre, P. Debyelaan 25, 6229 HX Maastricht, the Netherlands

**Keywords:** Core outcome set, Delphi, Endpoints, Hemorrhoids, Anal, Protocol

## Abstract

**Purpose:**

Over the last decade, many studies were performed regarding treatment options for hemorrhoidal disease. Randomised controlled trials (RCTs) should have well-defined primary and secondary outcomes. However, the reported outcome measures are numerous and diverse. The heterogeneity of outcome definition in clinical trials limits transparency and paves the way for bias. The development of a core outcome set (COS) helps minimizing this problem. A COS is an agreed minimum set of outcomes that should be measured and reported in all clinical trials of a specific disease. The aim of this project is to generate a COS regarding the outcome of treatment after hemorrhoidal disease.

**Methods:**

A Delphi study will be performed by an international steering group healthcare professionals and patients with the intention to create a standard outcome set for future clinical trials for the treatment of hemorrhoidal disease. First, a literature review will be conducted to establish which outcomes are used in clinical trials for hemorrhoidal disease. Secondly, both healthcare professionals and patients will participate in several consecutive rounds of online questionnaires and a face-to-face meeting to refine the content of the COS.

**Discussion:**

Development of a COS for hemorrhoidal disease defines a minimum outcome-reporting standard and will improve the quality of research in the future.

**Electronic supplementary material:**

The online version of this article (doi:10.1007/s00384-017-2833-5) contains supplementary material, which is available to authorized users.

## Background

Hemorrhoidal disease is one of the most common anorectal disorders presenting to coloproctological units, with a prevalence of 5–35% in the overall population [1, 2]. The true incidence of hemorrhoidal disease is difficult to estimate, as many patients are reluctant to seek medical therapy for various personal, cultural, and socioeconomic reasons. Hemorrhoids are enlarged vascular cushions in the anal canal, which can bleed because of both degenerative effects of aging and the repeated passage of hard stool [1]. People experience the following most common complaints: blood loss, prolapse, pain, itching, and pruritus. Conservative and/or medical treatment, including diet, lifestyle changes, and application of topical ointments, is often offered first [3]. When these treatment options fail, a common next treatment step is rubber band ligation (RBL) [4, 5]. Grade III and IV hemorrhoidal disease is often directly treated by more invasive surgical interventions including the sutured hemorrhoidopexy [6], the stapled hemorrhoidopexy [7, 8], the Doppler-guided hemorrhoidal artery ligation (DGHAL) [9–11], and the traditional hemorrhoidectomy [12, 13].

The ability to compare findings between studies and synthesize data in meta-analysis is limited because the outcomes are inconsistently defined and reported in clinical trials. This hampers interpreting treatment effect and making evidence-based healthcare decisions [14, 15].

The Outcome Measures in Rheumatology (OMERACT) [16] and Core Outcome Measures in Effective Trials (COMET) [17] (http://www.comet-initiative.org/) initiatives responded to the problem by developing a guideline for the creation of a core outcome set (COS).

This paper describes the study protocol developing a COS that should be considered mandatory for inclusion in all future clinical trials on treatment of hemorrhoidal disease.

## Methods/design

Development of a COS incorporates the Delphi methodology and exists of a stepwise approach [18]. The guideline on the usage of the Delphi technique and the checklist will be followed [19]. First, a systematic literature review will be performed to identify potential outcomes. Secondly, several rounds of online questionnaires will be conducted involving researchers and healthcare professionals.

### Search strategy

A broad-search strategy will be conducted to identify all published guidelines, reviews, systematic reviews, meta-analyses, and protocols regarding hemorrhoidal disease. The search will be limited to English language articles published in full-text. Searches will be performed in the following database: PubMed, EMBASE, and Cochrane between January 2012 and December 2016 (Appendix 1).

### Selection of articles

The review will be performed by two researchers (RT and JM) to identify all possible clinical outcomes reported in treatment studies for hemorrhoidal disease. First, the identified abstract will be screened. Studies where the outcomes are not treatment-related (e.g. health policy) will be excluded. In order to ensure accuracy of exclusion, a proportion of the excluded abstracts will be reviewed by a third reviewer (SB).

### Data extraction

For each trial, we will assess the author, date of publication, study design, compared treatments/interventions, primary and secondary outcomes, definition of these outcomes, and patient-reported outcomes (PROs).

### Participants

We will involve healthcare professionals and patients. We found no guidelines for the sample sizes of this study [18, 20]. However, having more stakeholders will increase the reliability of the group judgment [19, 20]. Healthcare professionals will be selected from a diverse range of international institutions and organizations. We will select healthcare professionals who have multiple cited publications in the field of proctology and/or who are familiar with the development of a COS. Further, we will use the “snowball sampling” approach. This method gives the healthcare professionals the opportunity to add someone they want to be included as panel member. We aim to recruit around 30 healthcare professionals by e-mail.

Patients will be invited to participate when they visit the outpatient clinic for the first time. Participants have to be diagnosed with hemorrhoidal disease and have to agree to participate in all Delphi rounds needed for this COS. We expect to recruit about 20 patients in total.

### Online survey

The list of outcomes, originating from the systematic review, will be formatted into questions with a Likert response design [21, 22], an evaluation scale from 1 to 9, to identify outcomes of importance to both healthcare professionals and patients. Questionnaires in the Delphi survey will be pilot tested by at least two members of the steering group (SB and LS).

Currently, there is no consensus in literature regarding the cut offs for inclusion and exclusion of outcomes in this process. Therefore, a flexible and pragmatic approach will be employed. “Consensus in” (information essential to the core set) will be defined as greater than 70% of participants scoring as 7–9 and less than 15% of participants scoring as 1–3. “Consensus out” (information should not be included in core set) will be defined as greater than 70% of participants scoring as 1–3 and less than 15% of participants scoring as 7–9. “Disagreement” will occur when 33% or more score 1–3 and 33% or more score 7–9 for a particular item. All other combinations will be considered “Equivocal,” which means that these items are open to more than one interpretation [23].

Over the course of several rounds, the expectation is that the range of items will decrease and the group converges toward a consensus opinion. This part of the process will end when the list of items is reduced to ten or less; or on completion of the second round voting [Appendix 2].

#### Round 1

In the first round, the panel members will be approached by a personalized e-mail with a link to a web-based survey (SurveyMonkey). Once participants have registered for the survey, names and email addresses will be stored in the system. This will allow identification of participants completing all rounds of the Delphi survey.

Initially, the panel members will be provided with one page background information on the rationale of the development of the COS. Then, they will be asked to list all items that they consider important or relevant in the COS. All items included in Delphi round 1 will be assigned to one of the four categories and “consensus in” and “consensus out” domains will be brought forward for the second round. Items designated “Disagreement” will undergo further analysis: mean scores will be calculated, and if the mean is above or below 5 (i.e., tending toward “consensus in” or “consensus out”), the item will be included in the second round. Items designated “Equivocal” will not be carried forward.

#### Round 2

The panel members from round 1 will then be invited to undertake round 2 of the process. During the second round, the stakeholders will be asked to review their initial responses in the light of groups’ responses [Appendix 3]. The responses of round 1 will be aggregated and send back to the panel members anonymously in a feedback report. The participants do not know the identities of other individuals in the panel group to make sure that the views of participants are obtained by a method that gives equal influence. This report will include the panel members’ ratings on the several questions/items, including the median scores, the interquartile ranges, their comments, and suggestions. Descriptive statistics summarizing the results of round 1 will be available for the panel members to review [Appendix 4]. Participants will also be provided with an option to add additional items that they think are missing.

#### Round 3

The third round will only be undertaken if significant numbers of outcomes remain in the short list after round 2. The results of round 2 will again be send back to all panel members in a feedback report. Then, the panel members will be asked to re-rate the items in view of the groups’ response.

### Consensus meeting

If consensus has not been reached after the three Delphi rounds or there is significant disagreement between the stakeholders, we will conduct a face-to-face meeting with the panel members initially invited.

By the end of the process, we should have identified “what” COS should be measured in clinical trials for hemorrhoidal disease. However, we may not be clear on “how” we should measure these.

## Discussion

The aim of this project is the development of a COS for hemorrhoidal disease. At the time of writing, there is no published COS for hemorrhoidal disease. The development of COS for the management of hemorrhoidal disease will facilitate evidence synthesis by reducing heterogeneity between trials.

The Delphi method [18, 24] is the appropriate instrument in decision-making processes in groups as feedback can be provided in a controlled anonymous manner. A major drawback is that the results of a Delphi study are highly dependent upon the composition of the panel and the selection of its members. To provide the highest possible input, we will include the healthcare professionals from a diverse range of international institutions and organizations.

We must actively engage with trial-lists and those that fund and publish trials to ensure that our COS is used in the rest of the academic world. With help from the panel members, we will identify how to reach relevant audience, including people using services, carers, the public, practitioners, and providers.

### Trial status

The Delphi study is currently ongoing. We are preparing to recruit participants to the Delphi study. The final COS is expected by the end of 2017.

## Electronic supplementary material

ESM 1 (DOCX 47 kb).
