# Supplementary material for: Protocol for the development of a Core Outcome Set (COS) for hemorrhoidal disease: an international Delphi study
Source: Int J Colorectal Dis. 2017 May 13;32(7):1091–4. doi: 10.1007/s00384-017-2833-5 (PMC5486628; doi:10.1007/s00384-017-2833-5)
Supplement: Supplementary file 1 — (DOCX 47 kb). [file 384_2017_2833_MOESM1_ESM.docx]

**Supplementary material**

**Appendix 1**

***Search strategies***

Pubmed:

| 1. #13,"Search (((((hemorrhoid* [Title/Abstract]) OR ((((((haemorrhoid*[Title/Abstract]) OR hemorhoid*[Title/Abstract]) OR haemorhoid*[Title/Abstract]) OR hemorroid*[Title/Abstract]) OR haemorroid*[Title/Abstract]) OR hemoroid*[Title/Abstract])) OR hemorrhoid[MeSH Terms])) AND ((((((((((((((surgery[MeSH Subheading]) OR surgery) OR surgical procedures, operative[MeSH Terms]) OR surgical) AND procedures) AND operative) OR operative surgical procedures) OR surgery) OR general surgery[MeSH Terms]) OR general) AND surgery) OR general surgery)) OR (((((treatment[MeSH Subheading]) OR therapy) OR treatment) OR therapeutics[MeSH Terms]) OR therapeutics))) AND (((((recurrence[MeSH Terms]) OR recurrence)) OR (((((diagnosis[MeSH Subheading]) OR diagnosis) OR symptoms) OR diagnosis[MeSH Terms]) OR symptoms)) OR (((((outcome assessment health care[MeSH Terms]) OR patient outcome assessment[MeSH Terms]) OR treatment outcome[MeSH Terms]) OR clinical effectiveness) OR treatment effectiveness))",2707,06:22:16 2. (#10 AND #11 AND #12) |
| --- |
| 1. #12,"Search ((((recurrence[MeSH Terms]) OR recurrence)) OR (((((diagnosis[MeSH Subheading]) OR diagnosis) OR symptoms) OR diagnosis[MeSH Terms]) OR symptoms)) OR (((((outcome assessment health care[MeSH Terms]) OR patient outcome assessment[MeSH Terms]) OR treatment outcome[MeSH Terms]) OR clinical effectiveness) OR treatment effectiveness)",9328185,06:21:54 2. (#7 OR #8 OR #9) |
| 1. #11,"Search (((((((((((((surgery[MeSH Subheading]) OR surgery) OR surgical procedures, operative[MeSH Terms]) OR surgical) AND procedures) AND operative) OR operative surgical procedures) OR surgery) OR general surgery[MeSH Terms]) OR general) AND surgery) OR general surgery)) OR (((((treatment[MeSH Subheading]) OR therapy) OR treatment) OR therapeutics[MeSH Terms]) OR therapeutics)",10554636,06:21:22 2. (#5 OR #6) |
| 1. #10,"Search ((hemorrhoid* [Title/Abstract]) OR ((((((haemorrhoid*[Title/Abstract]) OR hemorhoid*[Title/Abstract]) OR haemorhoid*[Title/Abstract]) OR hemorroid*[Title/Abstract]) OR haemorroid*[Title/Abstract]) OR hemoroid*[Title/Abstract])) OR hemorrhoid[MeSH Terms]",6602,06:21:03 2. (#2 OR #3 OR #4) |
| 1. #9,"Search ((((outcome assessment health care[MeSH Terms]) OR patient outcome assessment[MeSH Terms]) OR treatment outcome[MeSH Terms]) OR clinical effectiveness) OR treatment effectiveness",1197546,06:19:38 |
| 1. #8,"Search ((((diagnosis[MeSH Subheading]) OR diagnosis) OR symptoms) OR diagnosis[MeSH Terms]) OR symptoms",8982014,06:17:16 |
| 1. #7,"Search (recurrence[MeSH Terms]) OR recurrence",406696,06:16:22 |
| 1. #6,"Search ((((treatment[MeSH Subheading]) OR therapy) OR treatment) OR therapeutics[MeSH Terms]) OR therapeutics",9498187,06:16:05 |
| 1. #5,"Search (((((((((((surgery[MeSH Subheading]) OR surgery) OR surgical procedures, operative[MeSH Terms]) OR surgical) AND procedures) AND operative) OR operative surgical procedures) OR surgery) OR general surgery[MeSH Terms]) OR general) AND surgery) OR general surgery",4051959,06:15:19 |
| 1. #4,"Search hemorrhoid[MeSH Terms]",4871,06:12:15 |
| 1. #3,"Search (((((haemorrhoid*[Title/Abstract]) OR hemorhoid*[Title/Abstract]) OR haemorhoid*[Title/Abstract]) OR hemorroid*[Title/Abstract]) OR haemorroid*[Title/Abstract]) OR hemoroid*[Title/Abstract]",1761,06:11:16 |
| 1. #2,"Search hemorrhoid* [Title/Abstract]",4116,06:08:55 |

Cochrane:

1. #1, "hemorrhoid*":ti,ab,kw (Word variations have been searched)
2. #2, haemorrhoid*
3. #3, hemorrhoid*
4. #4, haemorroid*
5. #5, hemorroid*
6. #6, haemorhoid*
7. #7, hemorhoid*
8. #8, haemoroid*
9. #9, hemoroid*
10. #1 or #2 or #3 or #4 or #5 or #6 or #7 or #8 or #9
11. #11, surgery
12. #12, surgical procedures
13. #13, general surgery
14. #14, procedures
15. #15, treatment
16. #16, therapy
17. #17, therapeutics
18. #11 or #12 or #13 or #14 or #15 or #16 or #17
19. #19, recurrence
20. #20, outcome assessment
21. #21, treatment outcome
22. #19 or #20 or #21
23. #10 and #18 and #22

Embase:

1. #1, hemorrhoid*.mp. or exp hemorrhoid/

2. #2, haemorrhoid.mp. or exp hemorrhoid/

3. #3, hemorroid.mp.

4. #4, haemorroid.mp.

5. #5, hemoroid.mp.

6. #6, haemorhoid.mp.

7. #7, hemorhoid.mp.

8. #1 or #2 or #3 or #4 or #5 or #6 or #7

9. #9, exp general surgery/ or exp surgery/ or surgery.mp.

10. #10, surgical procedures.mp. or exp surgical technique/

11. #11, treatment.mp.

12. #12, exp therapy/ or therapy.mp.

13. #13, exp therapy/ or therapy.mp.

14. #9 or #10 or #11 or #12 or #13

15. #15, recurrence.mp. or exp recurrent disease/

16. #16, exp diagnosis/ or diagnosis.mp.

17. #17, exp symptom assessment/ or exp symptom/ or symptoms.mp.

18. #18, outcome assessment.mp. or exp outcome assessment/

19. #19, treatment outcome.mp. or exp treatment outcome/

20. #20, exp treatment outcome/ or exp outcome assessment/ or outcome.mp.

21. 15 or 16 or 17 or 18 or 19 or 20

22. #8 and #14

23. #21 and #22

**Appendix 2**

**Schema for the development of a Core Outcome Set**

Clinical systematic review

Long list of outcomes

Operationalisation into items for Delphi process

Delphi round 3

Delphi round 1

Delphi round 2

Consensus meeting

Core outcome set

**Appendix 3**

**Overview for Delphi round 2**

**Patients**

**Healthcare professionals**

**Healthcare professionals**

Receive feedback of scores from Delphi round 1 of other healthcare professionals for items included in round 2

**Patients and Healthcare professionals**

Receive feedback of scores from Delphi round 1 from both patients and healthcare professionals for items included in round 2

**Patients**

Receive feedback of scores from Delphi round 1 of other patients for items included in round 2

**Appendix 4**

**Analysis of questionnaire results**

| **Table A. MS Excel 2010 formulas used to assess appropriateness and disagreement.** | |
| --- | --- |
| Median of panel rating | =MEDIAN(x:x) |
| 30^th^ percentile | =PERCENTILE.EXC(x:x;0,3) |
| 70^th^ percentile | =PERCENTILE.EXC(x:x;0,7) |
| Interpercentile range 30^th^-70^th^ | =[70^th^ percentile]-[30^th^ percentile] |
| Central point IPR | =([70^th^ percentile]+[30^th^ percentile])/2 |
| Asymmetry Index | =ABS(5-[central point IPR]) |
| IPRAS | =2,35+(1,5*[Asymmetry index]) |
| IPRAS-IPR | =[IPRAS]-[IPR] |

Panel members score each item on a nine-point scale, where 1 equals “Very inappropriate” and 9 equals “Very appropriate”. Analysis of rating of events was conducted using MS Excel 2010. The formulas that were used are listed in Table A.

Consensus was defined as a panel median between 1 and 3 (Inappropriate) or between 7 and 9 (Appropriate) without disagreement. Inversely, this means that consensus did not exist if the panel median was between 4 and 6 (Uncertain), or if the answers varied so much that the definition of disagreement was met.

Disagreement was assessed according to the IPRAS Method as described in the RAND/UCLA Appropriateness Method Manual. Traditionally, the Appropriateness Method defined disagreement based on the amount of panel members that voted outside the 3-point range that contained the median. However, for panels consisting of more than nine members, another method, based on the InterPercentile Range (IPR), is recommended. A smaller IPR of the panel’s answers reflects more agreement. The 30^th^-70^th^ percentile range is used because it most accurately reflects the traditional RAND/UCLA definition of disagreement. However, the IPR in itself is not sufficient to assess agreement. One also needs to adjust for symmetry of the answers, because the IPR in itself does not take into account if the answers are at the same side of the rating scale of if there are extreme differences between panel members (reflected in answers distributed symmetrically on both sides of the rating scale). To illustrate the importance of correcting for symmetry, an example for a nine-member panel is shown in table B. The IPR is the same for both samples, although it is clear that panel members did not agree as much on question 1 as they did on question 2. To correct for this problem, RAND/UCLA developed a formula called IPRAS (InterPercentile Range Adjusted for Symmetry). In short, the IPRAS method determines if disagreement is present, based on the IPR of the ratings of the panel members, adjusted for symmetry.

| **Table B. Example of the difference between interpercentile range (IPR) and interpercentile range adjusted for symmetry (IPRAS) in a nine member panel.** | | | | | | | | | | | |
| --- | --- | --- | --- | --- | --- | --- | --- | --- | --- | --- | --- |
|  | **Panel ratings** | | | | | | | | | **IPR 30%-70%** | **IPRAS-IPR** |
|  | *a* | *b* | *c* | *d* | *e* | *f* | *g* | *h* | *i* |  | |
| Item 1 | *1* | *1* | *3* | *5* | *5* | *5* | *7* | *9* | *9* | 4 | -1,65: disagreement |
| Item 2 | *1* | *1* | *1* | *3* | *3* | *3* | *5* | *5* | *5* | 4 | 1,35: agreement |

The IPRAS formula (see Table A) contains fixed variables and a measure of asymmetry, the Asymmetry Index. As the answers of the panel (and therefore the symmetry of the answers) differ per item, every item has its own Asymmetry Index. The IPRAS reflects the broadest IPR that would constitute agreement at a certain Asymmetry Index. Next, the IPRAS can be compared to the actual IPR of the ratings of the panel. If the actual IPR is larger than the calculated IPRAS, this means disagreement is present taking into account the asymmetry of the answers. Therefore, IPRAS-IPR is <0 if the actual IPR of the ratings is larger than the range that would be the threshold for disagreement at the particular level of asymmetry of the answers. Thus, IPRAS-IPR indicates agreement if >0 and disagreement if <0.

In the example in Table B, scores on both items have a 30%-70% IPR of 4. For item 1, the calculated IPRAS minus the observed IPR results in disagreement, reflecting the fact that in question 1, panel members answered on both extremes of the scale, whereas in question 2, there was some uncertainty but answers were generally on the low side of the rating scale, which was recognized as in this case IPRAS-IPR does indicate agreement.
